# Retraction: Progesterone receptor antagonist provides palliative effects for uterine leiomyoma through a Bcl-2/Beclin1-dependent mechanism

**DOI:** 10.1042/BSR-2019-0094_RET

**Published:** 2024-11-15

**Keywords:** apoptosis, autophagy, mifepristone, progesterone receptor antagonist, progesterone receptor-M, uterine leiomyoma

This Retraction follows an Expression of Concern relating to this article previously published by Portland Press.

This article “Progesterone receptor antagonist provides palliative effects for uterine leiomyoma through a Bcl-2/Beclin1-dependent mechanism” DOI: 10.1042/BSR20190094 is being retracted from *Bioscience Reports* at the request of the Editor-in-Chief and the Editorial Board. This follows receipt of a notification from a reader, alerting the Editorial Board to:

- The Western blots, which appear to have minimal background details and share similarities with those published in papers by different authors
- Figure 2D oe-NC panel which seems to appear as Figure 2D MCF-7/EPI pharmorubicin (-) Cyto-ID+DAPI panel from Pei et al, 2018 (DOI: 10.1186/s12891-020-3159-y) with colour alteration
- Figure 4B Control panel which seems to appear as Figure 2D MCF-7/EPI pharmorubicin (+) Cyto-ID+DAPI panel from Pei et al, 2018 (DOI: 10.1186/s12891-020-3159-y) with colour alteration
- Figure 6C H-MIF panel which seems to appear as Figure 4C NC Overlay panel from He et al, 2019 (DOI: 10.18632/aging.101737) with colour and brightness alteration

The Editorial Office has additional concerns that the flow cytometry scatter graphs in Figure 4 and Figure 6 were modified during the revision process. The authors have been contacted with regards to the retraction and have not responded to the Journal's queries or the concerns raised. Given the extent of the issues raised, the Editorial Board stand by the decision to retract the article.

